# Analysis of corneal astigmatism and aberration in chinese congenital cataract and developmental cataract patients before cataract surgery

**DOI:** 10.1186/s12886-020-01794-2

**Published:** 2021-01-13

**Authors:** Xiaoyan Han, Qi Fan, Zhixiang Hua, Xiaodi Qiu, Dongjin Qian, Jin Yang

**Affiliations:** 1 Eye Institute and Department of Ophthalmology, Eye and Ear, Nose, and Throat Hospital, Fudan University, 200031 Shanghai, China; 2 The Key Laboratory of Myopia, Ministry of Health, 200031 Shanghai, China; 3 Shanghai Key Laboratory of Visual Impairment and Restoration, 200031 Shanghai, China; 4 Visual Rehabilitation Professional Committee, Chinese Association of Rehabilitation Medicine, 200031 Shanghai, China; 5 Department of Ophthalmology, Eye and ENT Hospital of Fudan University, 83 Fenyang Rd, 200031 Shanghai, China

**Keywords:** Congenital cataract, Developmental cataract, Astigmatism, Aberration, Category

## Abstract

**Background:**

To investigate the distribution of corneal astigmatism, aberration, and biometric parameters in Chinese congenital cataract (CC) /developmental cataract patients before cataract surgery.

**Methods:**

We evaluated eyes of CC /developmental cataract patients scheduled for cataract surgery from January 2016 to September 2019. Astigmatism, aberrations, and biometric parameters were measured with the Pentacam Scheimpflug imaging device (Pentacam HR, Oculus). Cataract was diagnosed and classified by slit-lamp examination after full mydriasis.

**Results:**

We evaluated 538 eyes in 356 patients. The mean values of anterior corneal astigmatism (ACA), posterior corneal astigmatism (PCA), and total corneal astigmatism (TCA) were determined as 1.98 ± 1.06 D (range 0.0‒4.8 D), 0.49 ± 0.26 D (range 0.0‒1.9 D), and 2.09 ± 1.19 D (range 0.2‒8.8 D), respectively. ACA and TCA ≥ 1.25 D was present in 379 eyes (70.3%) and 392 eyes (72.8%), respectively. PCA between 0.25 D and 0.75 D was found in 380 eyes (70.6%). There was a statistically significant positive linear correlation between lower-order aberrations root mean square values (LOA RMS) and corneal astigmatism (CA). Furthermore, in terms of distribution of central cornea thickness, anterior chamber depth, ACA, PCA, and TCA in different types of cataracts, ACA was highest in patients with zonular cataracts. Finally, we found anterior corneal measurements may overestimate WTR astigmatism, underestimate ATR astigmatism, and underestimate oblique astigmatism, respectively.

**Conclusions:**

Most CC /developmental cataract patients had moderate to high astigmatism and ACA accounted for the largest proportion in the zonular group. This can provide a basis for planning of CC/developmental cataract surgery by ophthalmologists in clinical practice.

## Background

Congenital cataract (CC) /developmental cataract is the main cause of visual impairment and blindness in children around the world [1, 2]. There is still no standardized classification of CC/developmental cataract due to the complicated presentations of lens opacities, although it can be clinically categorized into several groups according to the etiology, anatomic location, or shape of the lens opacity [3–5].

With the advancement of phacoemulsification technology and equipment, cataract surgery has changed from rehabilitative surgery to refractive surgery. Astigmatism is a refractive error; it includes corneal astigmatism (CA) and internal astigmatism, of which the former is the main source of astigmatism in the optical system. Astigmatism affects various visual functions, such as visual acuity and contrast sensitivity [6–8]. For cataract patients with CA, several studies have shown that implantation of a toric intra-ocular lens (IOL) can greatly improve postoperative visual quality [9, 10]. Correspondingly, there appears to be increasing requirements for preoperative ophthalmic examinations, in particular, accurate CA examinations. Therefore, it is increasingly important to know the distribution of astigmatism, such as anterior corneal astigmatism (ACA), posterior corneal astigmatism (PCA), and total corneal astigmatism (TCA), as well as aberrations in patients. Lin et al. previously reported the distribution of CA and anterior segment biometry in CC patients and showed that different types of lens opacities may be associated with anterior segment abnormalities [11]. However, no studies to date have illustrated the distribution of ACA, PCA, TCA, and aberrations comprehensively. Moreover, although there is consensus that toric IOLs can be implanted to correct astigmatism in age-related cataract (ARC) patients [9, 10], for CC/developmental cataract patients, particularly for younger children, the feasibility and timing of toric IOL implantation remain controversial, as CA changes with age.

Therefore, in our study, we investigated the distribution of ACA, PCA, TCA, aberrations, central corneal thickness (CCT) and anterior chamber depth (ACD) before surgery in Chinese CC/developmental cataract patients. Moreover, these parameters were further evaluated in different CC/developmental cataract types to identify their relationship, in order to provide a theoretical basis for clinicians to treat CC/developmental cataract patients accurately by cataract surgery.

## Methods

From January 2016 to September 2019, this retrospective study was carried out by recruiting patients, from all over the country, scheduled for cataract surgery at the Eye and ENT Hospital of Fudan University, Shanghai, one of the largest ophthalmology specialist hospitals in China. Participants were eligible if the patients were diagnosed with CC/developmental cataract and older than 5 years before surgery and could cooperate well with the ophthalmic examinations. Eyes with corneal and iris diseases, lens subluxation, childhood glaucoma, retinal and choroid diseases, nystagmus and nanophthalmos were excluded. The study was approved by the Human Ethics Committee of the Eye and ENT Hospital of Fudan University. All procedures adhered to the tenets of the Declaration of Helsinki and written informed consent was obtained from the patients or guardians of all participants.

The Pentacam HR system (Oculus Inc., Wetzlar, Germany), a 3-dimensional anterior segment imaging and analysis system, was used to measure biometric parameters, including CCT, ACD, ACA, PCA, TCA, and aberrations. Furthermore, a 6.0 mm pupil scan diameter was performed to evaluate optical aberrations. All CC/developmental cataract patients were examined by a qualified and experienced doctor and tropicamide eye drops were administered to obtain full mydriasis before surgery. The software of this device was able to correct distortions in the Scheimpflug images based on the geometry of the Scheimpflug principle, which provides good data qualification.

For a comprehensive analysis, astigmatism was classified into with-the-rule (WTR), against-the-rule (ATR), and oblique astigmatism. For ACA and TCA, when the steep meridian of the corneal surface was between 60° and 120°, it was defined as WTR, and when the steep meridian of the corneal surface was 0–30° or 150–180°, it was defined as ATR. However, because of the opposite corneal surface curve, PCA was defined as WTR when the steep meridian was 0–30° or 150–180°, or as ATR when the steep meridian was 60‒120°. Any other astigmatism was defined as oblique astigmatism.

Moreover, astigmatism was divided by degree into three groups: low (< 1.25 D), moderate (1.25 to 2.750 D), and high (larger than 2.75 D). Then, the total corneal root mean square (TRMS), LOA RMS, and higher-order aberrations RMS (HOA RMS) for the anterior, posterior, and total cornea were recorded for further analysis.

Mean and standard deviation (mean ± SD) were used to analyze quantitative variables. Absolute number (n) and frequency (%) were used to analyze qualitative variables. The normal distribution of all variables was evaluated by the Kolmogorov‒Smirnov test. Wilcoxon test were used to analyze the non-normally distributed data, and χ2 test and Fisher’s exact test were used to compare categorical items. Comparisons between more than two groups were performed by Kruskal-Wallis test. Pearson’s correlation and regression analysis were used to analyze the relationships between PCA and age, between PCA and ACA, and between CA and aberrations. Statistical significance was assumed when p < 0.05. All data analysis was conducted using SPSS Statistics version 26.0 (IBM/SPSS, Inc., Chicago, IL).

## Results

**Fig. 1:**
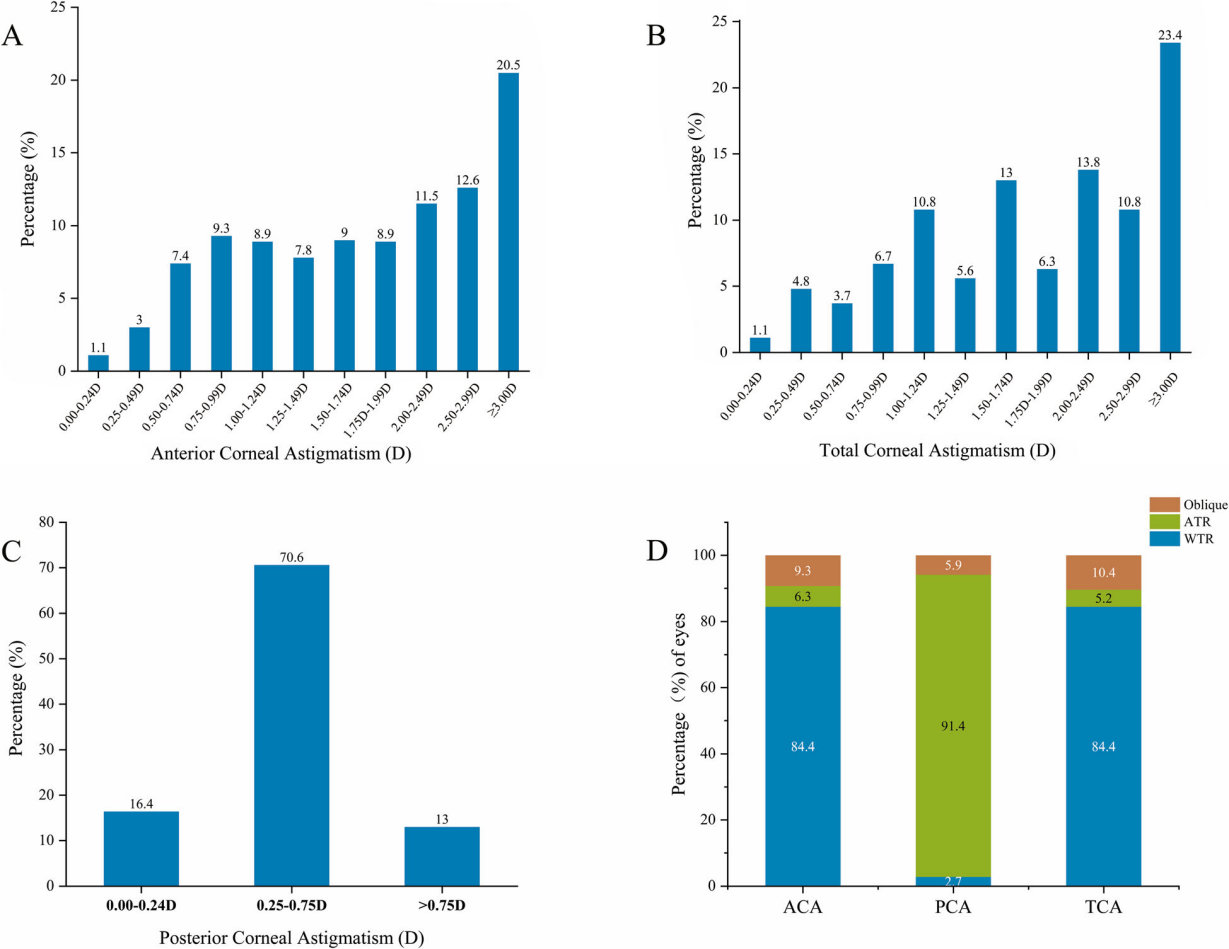
Distribution of astigmatism. **a** Distribution of anterior corneal astigmatism (ACA) in all congenital cataract (CC) patients. **b** Distribution of total corneal astigmatism (TCA) in all CC patients. **c** Distribution of posterior corneal astigmatism (PCA) in all CC patients. **d** The overall distribution of ACA, PCA, and TCA in all CC patients

**Fig. 2:**
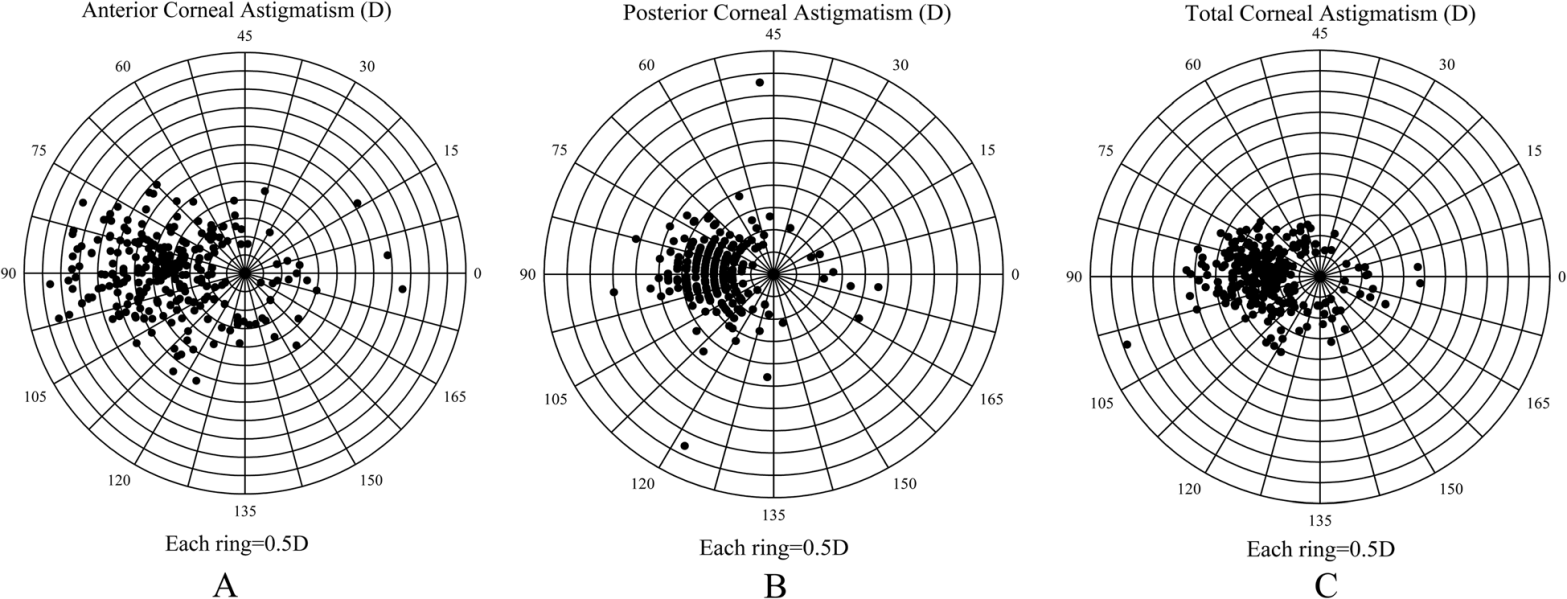
Double-angle plots of astigmatism. **a** Double-angle plots of anterior corneal astigmatism. **b** Double-angle plots of posterior corneal astigmatism. **c** Double-angle plots of total corneal astigmatism

**Fig. 3:**
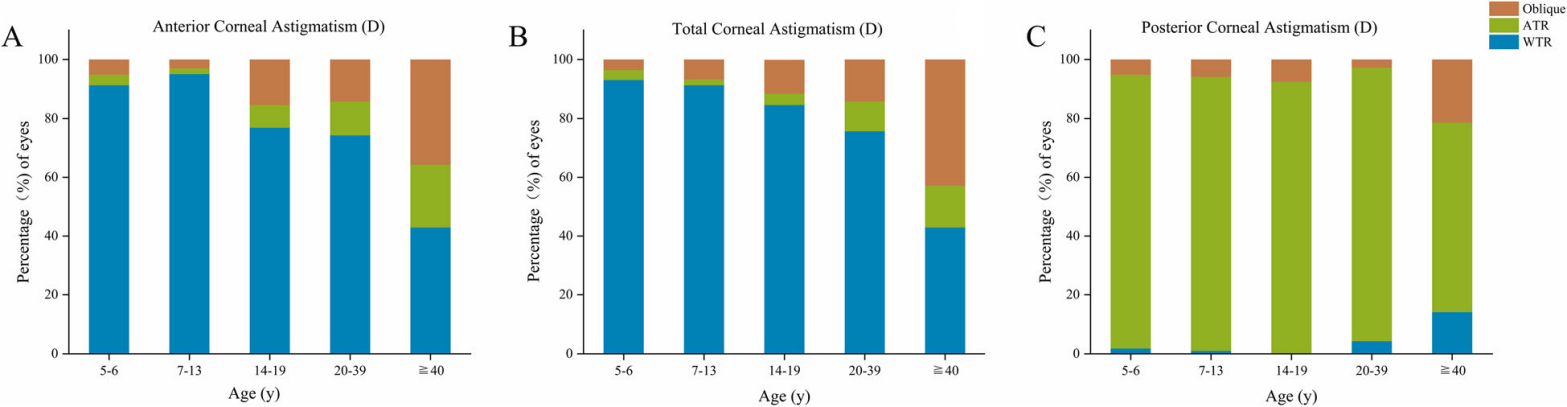
Percentage of eyes with WTR, ATR and oblique in each age on anterior corneal astigmatism (**a**), total corneal astigmatism (**b**) and posterior corneal astigmatism (**c**)

In total, 538 eyes (356 CC/developmental cataract patients, 290 males and 248 females) were enrolled in this study. The population demographics and the mean values of ACA, PCA, TCA, anterior corneal RMS (ARMS), posterior corneal RMS (PRMS), and TRMS are shown in Table 1. Furthermore, moderate and high astigmatism in ACA accounted for 242 eyes (45.0%) and 136 eyes (25.3%), while moderate and high astigmatism in terms of TCA was found in 246 eyes (45.7%) and 146 eyes (27.1%), respectively (Fig. 1a and b). In PCA, astigmatism from 0.25 D to 0.75 D was seen in 380 eyes (70.6%) (Fig. 1c). WTR astigmatism occurred mostly in those with ACA and TCA, although ATR astigmatism predominated in those with PCA (Fig. 1d). Furthermore, a double-angle plot was generated to show the astigmatism values and axis of ACA, PCA, and TCA more graphically (Fig. 2). Then, we further described ACA, PCA, and TCA in patients by age (Table 2). In ACA, WTR predominated within different age stages, and the percentage of WTR decreased gradually with age in 14‒19-year-olds, 20‒30-year-olds, and 40‒45-year-olds (*P* < 0.001). However, the percentage of ATR and oblique astigmatism showed an increasing tendency with age (*P* < 0.001) (Fig. 3a). The TCA had a similar tendency to ACA (*P* < 0.001) (Fig. 3b). In contrast, ATR astigmatism dominated in every age group with PCA, but the ATR proportion decreased with age (*P* < 0.001) (Fig. 3c).

**Table 1:** Preoperative patient characteristics

Characteristic	Male	Female	Total	*P* value
Age(y)				
Mean ± SD	15.84 ± 12.60	18.49 ± 13.26	17.06 ± 12.95	<0.001*
Range	5‒43	5‒45	5‒45	
Eyes (n)	290	248	538	‒
Sex (%)	52.8	47.2	100	‒
ACD^₮1^ (mm)				
Mean ± SD	3.67 ± 0.46	3.60 ± 0.46	3.63 ± 0.46	.573
Range	2.32‒5.03	2.00‒5.28	2.00‒5.28	
CCT^₮2^ (um)				
Mean ± SD	543.50 ± 36.12	536.54 ± 38.45	540.24 ± 37.31	.115
Range	448‒542	472‒532	448‒652	
ACA^₸1^ (D)				
Mean ± SD	1.95 ± 1.07	2.02 ± 1.06	1.98 ± 1.06	.741
Range	0.0‒4.4	0.1‒4.8	0‒4.8	
PCA^₸2^(D)				
Mean ± SD	0.50 ± 0.29	0.47 ± 0.22	0.49 ± 0.26	.631
Range	0.0‒1.9	0.0‒1.3	0.0‒1.9	
TCA^₸3^ (D)				
Mean ± SD	2.08 ± 1.24	2.08 ± 1.14	2.09 ± 1.19	.993
Range	0.2‒8.8	0.2‒5.4	0.2‒8.8	
ARMS^§1^ (um)				
Mean ± SD	2.81 ± 0.97	2.91 ± 1.02	2.86 ± 0.99	.420
Range	1.14‒5.32	0.98‒6.69	0.98‒6.69	
PRMS^§2^ (um)				
Mean ± SD	0.9 ± 0.41	0.93 ± 0.20	0.93 ± 0.33	.253
Range	0.47‒4.75	0.42‒1.62	0.42‒4.75	
TRMS^§3^ (um)				
Mean ± SD	2.40 ± 0.87	2.46 ± 0.95	2.43 ± 0.90	0.562
Range	0.78‒4.55	0.59‒5.81	0.59‒5.81	

*D* Diopter,^₮1^*ACD* Anterior Chamber Depth,^₮2^*CCT* Central Corneal Thickness, ^₸1^*ACA* Anterior Corneal Astigmatism, ^₸2^*PCA* Posterior Corneal Astigmatism, ^₸3^*TCA* Total Corneal Astigmatism, ^§1^*ARMS* Anterior corneal RMS, ^§2^*PRMS* Posterior corneal RMS, ^§3^*TRMS* Total corneal RMS

**Table 2:** The distribution of ACA, PCA, and TCA in different age groups

Characteristics	≤ 6 years	7‒13 years	14‒19 years	20‒39 years	≥ 40 years	*P* value
Eyes (n)	114	204	52	140	28	
ACA^₸1^ (D)						
Mean ± SD	2.13 ± 0.97	2.04 ± 1.07	2.35 ± 1.21	1.72 ± 0.96	1.49 ± 1.26	.002*
Range	0.5‒4.8	0.0‒4.4	0.5‒4.7	0.1‒3.8	0.4‒3.8	
PCA^₸2^(D)						
Mean ± SD	0.54 ± 0.31	0.51 ± 0.28	0.52 ± 0.21	0.42 ± 0.19	0.38 ± 0.28	.008*
Range	0.2‒1.9	0.0‒1.9	0.3‒1.0	0.0‒0.9	0.1‒0.9	
TCA^₸3^ (D)						
Mean ± SD	2.24 ± 0.95	2.17 ± 1.11	2.22 ± 1.35	1.81 ± 1.34	1.59 ± 1.27	.001*
Range	0.7‒4.8	0.2‒5.1	0.4‒5.4	0.2‒8.8	0.2‒3.9	

^₸1^*ACA* Anterior Corneal Astigmatism, ^₸2^*PCA* Posterior Corneal Astigmatism, ^₸3^*TCA* Total Corneal Astigmatism

**Fig. 4:**
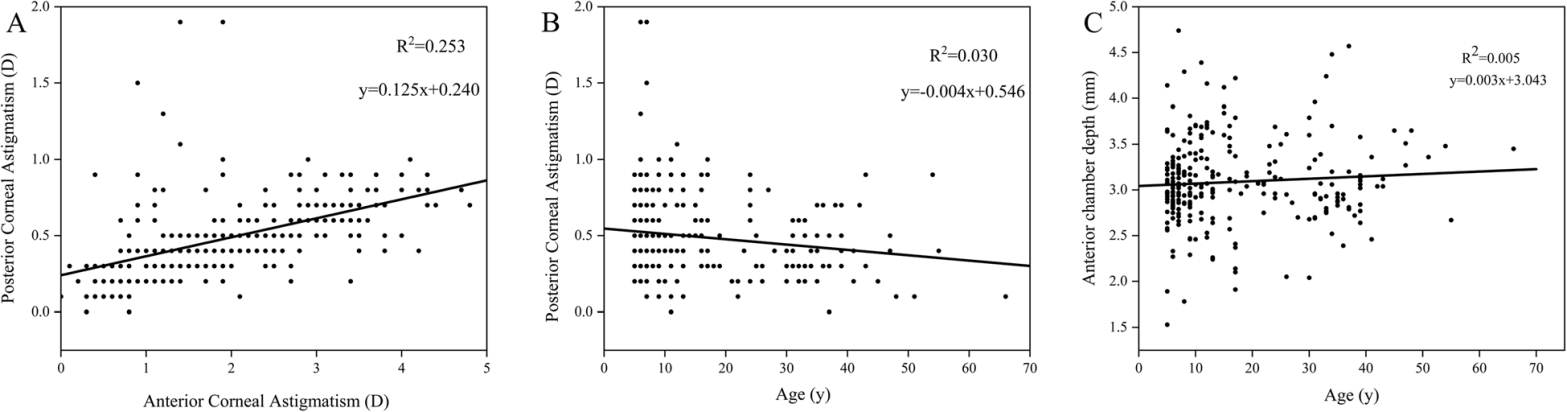
Correlation between posterior corneal astigmatism and anterior corneal astigmatism (**a**), between posterior corneal astigmatism and age, (**b**) and between anterior chamber depth and age (**c**)

**Fig. 5:**
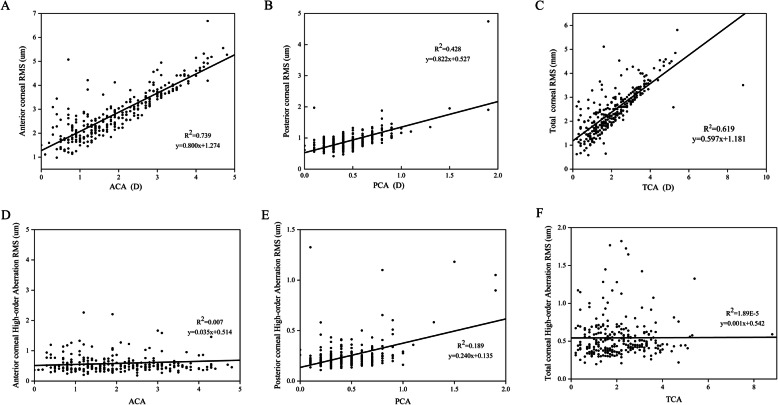
Correlation between three corneal astigmatism and aberration. **a** Correlation between ACA and anterior corneal RMS. **b** Correlation between PCA and posterior corneal RMS. **c** Correlation between TCA and total corneal RMS. **d** Correlation between ACA and anterior corneal HOA RMS. **e** Correlation between PCA and posterior corneal HOA RMS. **f** Correlation between TCA and total corneal HOA RMS. (ACA = anterior corneal astigmatism; PCA = posterior corneal astigmatism; TCA = total corneal astigmatism; HOA = high-order aberration; RMS = root mean square)

**Fig. 6:**
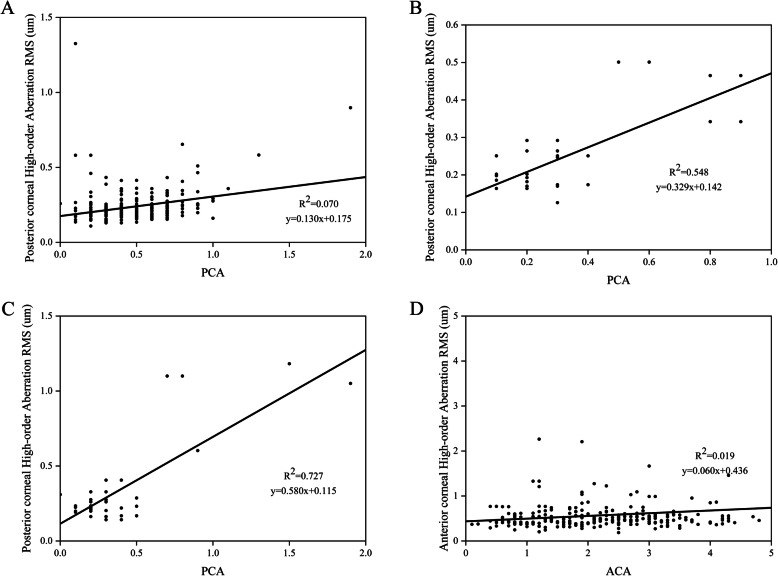
Correlation between corneal astigmatism and aberration. **a** Correlation between PCA and posterior corneal HOA RMS in eyes with WTR anterior corneal astigmatism. **b** Correlation between PCA and posterior corneal HOA RMS in eyes with ATR anterior corneal astigmatism. **c** Correlation between PCA and posterior corneal HOA RMS in eyes with Oblique anterior corneal astigmatism. **d** Correlation between ACA and anterior corneal HOA RMS in eyes with WTR anterior corneal astigmatism. (ACA = anterior corneal astigmatism; PCA = posterior corneal astigmatism; HOA = high-order aberration; RMS = root mean square)

There was a positive linear correlation (*R*^2^ = 0.253, *P* < 0.001) between PCA and ACA in all patients (Fig. 4a). A negative correlation was also found between PCA and age (*R*^2^ = 0.030, *P* < 0.005) (Fig. 4b), and there was no significant correlation between ACD and age (*R*^2^ = 0.005, *P* > 0.1) (Fig. 4c). Furthermore, a positive correlation was found between ACA and the ARMS (*R*^2^ = 0.739, *P* < 0.001). A similar correlation was found for PCA and PRMS (*R*^2^ = 0.428, *P* < 0.001), and for TCA and TRMS (*R*^2^ = 0.619, *P* < 0.001) (Fig. 5a‒c). Then, we analyzed the relationship between astigmatism and higher order aberrations (HOA). Analysis of the CA and HOA revealed that a significant positive linear correlation was shown between the PCA and posterior corneal HOA RMS [*R2* = 0.189, *P* < 0.001]. However, ACA and anterior corneal HOA RMS, TCA and total corneal HOA RMS showed no significantly statistical difference (Fig. 5d‒f). After classifying ACA into WTR, ATR and oblique group, we further analyzed the relationship between CA and HOA. Interestingly, we found the relationship of CA and HOA in ATR and oblique groups were consistent with the unclassified astigmatism analysis. The significantly linear correlation existed between PCA and posterior corneal HOA in WTR, ATR and oblique groups of ACA (Fig. 6a-c). Although the TCA and total corneal HOA RMS in WTR still showed no significantly statistical difference, the ACA and anterior corneal HOA RMS in WTR showed a significant positive correlation [*R2* = 0.019, *P* < 0.001] (Fig. 6d).

Furthermore, according to the location and shape of opacity, we classified CC/developmental cataract participants with typical opacity into five types (364 eyes, 266 patients), namely, zonular, nuclear, cortical, and posterior polar cataract. We next investigated the ACD, CCT, ACA, PCA, and TCA in these five groups (Table 3). We found that only ACA was statistically significantly differently distributed among the five groups, and was highest in patients in the zonular group. The ACA values decreased gradually through the nuclear, cortical, posterior polar, and total cataract groups.

**Table 3:** The distribution of ACD, CCT, ACA, PCA and TCA in five groups

Characteristics	Total	Zonular	Nuclear	Cortical	Posterior polar	*P* value
Eyes (n)	14	32	78	116	124	
ACD^₮1^ (mm)						
Mean ± SD	2.93 ± 0.85	3.21 ± 0.56	3.06 ± 0.42	3.00 ± 0.44	3.13 ± 0.37	.159
Range	1.53‒3.69	2.52‒4.48	2.39‒4.24	1.91‒4.39	1.89‒3.84	
CCT^₮2^ (um)						
Mean ± SD	552.50 ± 58.09	530 ± 34.00	548.43 ± 35.05	545.24 ± 35.95	533.60 ± 29.98	.261
Range	493‒634	462‒579	488‒652	479‒643	474‒611	
ACA^₸1^ (D)						
Mean ± SD	1.62 ± 0.93	2.55 ± 1.17	2.27 ± 1.08	1.86 ± 1.06	1.78 ± 0.80	.021*
Range	0.60‒3.10	0.10‒4.30	0.60‒4.40	0.30‒4.80	0.50‒4.00	
PCA^₸2^(D)						
Mean ± SD	0.50 ± 0.34	0.48 ± 0.19	0.54 ± 0.20	0.44 ± 0.28	0.51 ± 0.31	.164
Range	0.10‒0.90	0.2‒0.90	0.20‒1.00	0.00‒1.90	0.10‒1.90	
TCA^₸3^ (D)						
Mean ± SD	1.74 ± 0.88	2.69 ± 1.28	2.26 ± 1.20	1.92 ± 1.18	1.95 ± 0.84	.062
Range	0.80‒3.60	0.20‒5.40	0.40‒5.10	0.30‒5.20	0.40‒4.70	

Finally, compared with TCA, anterior corneal measurements overestimated WTR astigmatism by a mean of 0.26 ± 0.53 (D) in 57.7`% eyes, underestimated ATR astigmatism by 0.13 ± 0.16 (D) in 47.16% eyes, and underestimated oblique astigmatism by 0.33 ± 0.33 (D) in 72.0% eyes. The results were shown in Table 4.

**Table 4:** Anterior corneal astigmatism-estimated total corneal astigmatism distribution

Type of ACA	Overestimated	Underestimated		No influence		Total
	N (%)	Mean± SD (D)	N (%)	Mean± SD (D)	N (%)	N (%)
WTR	262 (57.71)	0.26±0.53	160 (35.24)	0.22±0.16	32 (7.05)	454 (100)
ATR	10 (29.41)	0.18±0.18	16 (47.06)	0.13±0.16	8 (23.53)	34 (100)
Oblique	12 (24.00)	0.18±0.12	36 (72.00)	0.33±0.33	2 (4.00)	50 (100)
Total	284 (52.79)	0.25±0.51	212 (39.41)	0.23±0.20	42 (7.80)	538 (100)

*ACA* Anterior Corneal Astigmatism, *WTR* With-the-rule, *ATR* against-the-rule

## Discussion

Having a good grasp of the details of CA in CC/developmental cataract patients can provide considerable guidance for cataract surgery and thus improve patient’s postoperative visual quality. However, few studies have described ACA, PCA, and TCA in a large number of cases of CC/developmental cataract patients, perhaps due to the low incidence of CC/developmental cataract, the few outpatients in local hospitals, the ignorance of such patients, and related data collecting, and the lack of appropriate equipments to examine CA accurately. The Pentacam HR system, a 3-dimensional anterior segment imaging and analysis system, was used in the current study, as it could provide the comprehensive parameters necessary for this study. In our study, more than 377 eyes (70%) with ACA and TCA had astigmatism ≥ 1.25 D, but astigmatism in PCA ranged mostly from 0.25 D to 0.75 D in 380 eyes (70.6%). Lin et al. found that the ACD of the cataractous eye showed a logarithmic relationship with age and the CCT of boys was thicker than that of girls with CC [2], and yet similar results were not found in our study. The possible reason was that we included a larger distribution of age in CC/developmental cataract patients (5–45 years old). To the best of our knowledge, no previous study had shown that most CC/developmental cataract patients have moderate to high astigmatism. Therefore, implantation of an appropriate toric IOL can greatly improve postoperative visual quality, and the therapeutic effect of amblyopia can also be augmented in children with CC/developmental cataract at a young age.

Previous findings have reported that CC/developmental cataract patients usually have quite high astigmatism and the mean CA in CC/developmental cataract patients exceeded 2 D [2, 5, 12]. Our results were similar to these studies, with a mean TCA of 2.09 ± 1.19 D. Nonetheless, there is a paucity of data on PCA in CC /developmental cataract patients. To our knowledge, our study was the first to investigate the distribution of ACA and PCA characteristics in Chinese CC/developmental cataract patients, particularly PCA. Moreover, Read and Vitalyos reported that CA could change with age and that PCA decreased significantly with age in ARC patients [6, 13]. In the present study, there was also a negative correlation between PCA and age. Several studies of ARC patients manifested that the mean magnitude of PCA commonly ranged between 0 and 1.0 D [14–16]. However, PCA values of CC/developmental cataract patients in our findings were larger than 1.0 D, which may be due to the large age span of CC /developmental cataract patients in this study. Moreover, previous studies have also illustrated that the ACA and TCA shifted from WTR to ATR with increasing age, but that PCA essentially remained ATR in the general population [17]. The results in the present study was consistent with those findings. Additionally, we found WTR astigmatism occurred mostly in those with ACA and TCA, although ATR astigmatism predominated in those with PCA. Therefore, the age-related corneal astigmatical may result in a low WTR proportion in ACA and TCA group, and we ought to take this effect into account when treating CC patients. Furthermore, this study found that, in CC/developmental cataract patients older than 14 years, the percentage of ATR astigmatism in those with ACA and TCA gradually increased. Therefore, considering that the ATR proportion increased with age, undercorrecting WTR astigmatism while overcorrecting ATR astigmatism appropriately might be suggested if a toric IOL was implanted for CC/developmental cataract patients younger than 14 years. Our study further found that a significant positive correlation existed between ACA and PCA, which implied that high ACA values were usually accompanied by high PCA values, which consequently has effect on the TCA. Hence, the effect of PCA cannot be neglected for CC/developmental cataract patients with moderate and high astigmatism when implanting a toric IOL.

A significantly positive linear correlation was found between ACA and ARMS in our study and similar results were found for PCA and PRMS, and TCA and TRMS. This meant that the higher CA corresponded with higher aberrations in CC/developmental cataract patients. According to the Zernike polynomials, aberration can be divided into LOA and HOA aberrations. After analyzing the LOA CA and HOA, a significant positive linear correlation was shown between the PCA and posterior corneal HOA RMS. Furthermore, we found a positive linear correlation existed between PCA and posterior corneal HOA in WTR, ATR and oblique groups of ACA (Fig. 6a-c). Besides, the ACA and anterior corneal HOA RMS showed a significant positive correlation in WTR groups of ACA. More importantly, no previous study had analyzed the relationship between astigmatism and aberration in CC patients; this was similar to the results found in ARC patients [17].

Furthermore, a previous prospective study defined a novel congenital cataract category system based on lens opacity locations and relevant anterior segment characteristics, and it also studied the CA and ACD in various cataract types [11]. However, the above classifications are rare in clinical practice, and thus, we divided CC into five types according to the location and shape of opacity: total, zonular, nuclear, cortical, and posterior polar cataract in our study, as these classifications are more commonly used. The ACA, PCA, TCA, ACD, and CCT were analyzed in these five groups. Interestingly, the ACA values of zonular cataracts were the highest among the five groups, and decreased sequentially in the nuclear, cortical, posterior polar, and total cataract groups. However, TCA did not show a statistically significant difference among the different types of C/developmental cataract C patients, which might be because the PCA in this study was higher, and then influenced TCA to some extent. In terms of ACA differences among different groups, we speculated that different optical pathways were produced by various types of lens opacities and then affected the development of the eye, particularly the cornea, which eventually led to CA changes [6, 18–21]. Moreover, zonular cataracts are generally more uneven and accompanied by more scatter-light, resulting in higher ACA than in other groups, due to visual feedback. Additionally, further studies are necessary to explore other possible mechanisms. Based on our results, insights have been gained on the relationship between the locations of lens opacities and anterior segment parameters in CC /developmental cataract patients. For CC /developmental cataract patients who do not cooperate well with examination equipment for the anterior segment in particular, our findings can assist ophthalmologists in speculating about possible astigmatism abnormalities, based on lens opacities that can be examined by slit-lamp microscopy, and then provides an efficient clinical guideline for CC/developmental cataract diagnosis and treatment.

Previous studies showed that ignoring posterior corneal astigmatism may yield significant estimation errors for total corneal astigmatism in age-related cataract patients preparing for cataract surgery. To be more specific, error in magnitude was significantly increased when the magnitude of posterior corneal astigmatism was larger than 0.4 D. Additionally, when WTR anterior corneal astigmatism was more than 2.6 D or an ATR astigmatism was more than 1.6 D, the anterior measurement can cause errors influencing on the toric IOL decision [15, 22]. In the present study, the posterior corneal astigmatism (PCA) of larger than 0.4 D, WTR anterior corneal astigmatism of larger than 2.6 D and ATR astigmatism of larger than 1.6 D accounted for 49.8, 32.2 and 23.5%, respectively. Besides, we found anterior corneal measurements may overestimate WTR astigmatism, underestimate ATR astigmatism, and underestimate oblique astigmatism, respectively. These results above revealed that PCA ought to be taken into consideration for CC/developmental cataract patients in preparing toric IOL implantation.

However, our study had some limitations. First, when we analyzed the different types of lens opacities, we excluded some eyes with quite complex morphologic characteristics that were difficult to classify, which caused the sample size in groups to be inadequate. Second, our study was conducted only by Pentacam, which might cause systematic variations in results; although statistical difference of ACA between zonular cataract and other groups did exist, it may be due to the relatively small number of samples in this group, which needs to be carefully considered. we will corroborate our measuring results by other corneal imaging methods in future. Third, our study was not a prospective study with long-term follow-up of the same patients; hence, it was impossible to analyze the changes of various astigmatism parameters, such as ACA, TCA, and PCA with age, systematically. Notwithstanding these limitations, the present study investigated three CA parameters in Chinese CC/developmental cataract patients in an expanded age range, which has not been reported previously. In addition, we analyzed the correlation between astigmatism and aberrations and explored the correlation between biometric parameters and different types of lens opacities. Consequently, these findings might greatly inform clinical practice of CC/developmental cataract diagnosis and treatment.

## Conclusions

The findings of this study suggested that most patients with CC/developmental cataract present moderate to high astigmatism, and timely implantation of toric IOLs should be given priority in CC/developmental cataract patients. Moreover, our study filled the gap in knowledge about PCA in CC/developmental cataract patients and propose that the effect of PCA should be considered when planning toric IOL in CC/developmental cataract patients with moderate and high CA. We found that ACA was highest in the zonular group. This information can provide a rough evaluation of astigmatism according to the type of cataract when patients cannot cooperate well with CA examination, which can could facilitate a more accurate choice of IOL type. Additionally, neglection of PCA is likely to overestimate the TCA in WTR anterior corneal eyes and underestimate the TCA in ATR and oblique anterior corneal eyes. Therefore, PCA ought to be taken into consideration for CC/developmental cataract patients who prepare to implant the toric IOL.

## Data Availability

The datasets used and/or analysed during the current study are available from the corresponding author on reasonable request.

## Abbreviations

CC: Congenital cataract
CA: Corneal astigmatism
ACA: Anterior corneal astigmatism
PCA: Posterior corneal astigmatism
TCA: Total corneal astigmatism
ARC: Age-related cataract
IOL: Intra-ocular lens
CCT: Central corneal thickness
ACD: Anterior chamber depth
WTR: With-the-rule
ATR: Against-the-rule
LOA RMS: Lower-order aberrations root mean square values
HOA RMS: Higher-order aberrations RMS
ARMS: Anterior corneal RMS
PRMS: Posterior corneal RMS
TRMS: Total corneal root mean square
